# The Microbiome in Childhood Acute Lymphoblastic Leukemia

**DOI:** 10.3390/cancers13194947

**Published:** 2021-09-30

**Authors:** Marina Oldenburg, Nadine Rüchel, Stefan Janssen, Arndt Borkhardt, Katharina L. Gössling

**Affiliations:** 1 Department of Pediatric Oncology, Hematology and Clinical Immunology, Medical Faculty, Center of Child and Adolescent Health, Heinrich-Heine-University, 40225 Düsseldorf, Germany; Marina.Oldenburg@med.uni-duesseldorf.de (M.O.); nadine.ruechel@med.uni-duesseldorf.de (N.R.); Arndt.Borkhardt@med.uni-duesseldorf.de (A.B.); 2 Algorithmic Bioinformatics, Department of Biology and Chemistry, Justus Liebig University Gießen, 35390 Gießen, Germany; stefan.janssen@computational.bio.uni-giessen.de

**Keywords:** ALL, oral microbiome, fecal microbiome, microbiota, leukemogenesis, infection-triggered leukemia, childhood leukemia, induction therapy, consolidation therapy, maintenance therapy

## Abstract

**Simple Summary:**

The role of the microbiome for the development and treatment of acute lymphoblastic leukemia (ALL) is not well understood. The immune system and microbiota closely interact and perturbations have strong implications for ALL development and course of the treatment. Significant differences in the microbiome with reduced diversity have been observed already at the onset of disease and have potential implications for leukemogenesis. Furthermore, the regular chemotherapeutic treatment regimen severely perturbs the microbiome, being associated with severe side effects such as mucositis, systemic inflammation, or infections. Herein, we review the latest microbiome studies in pediatric ALL patients, as well as provide an overview of current and future options to modulate the microbiome to improve the treatment’s outcome or even prevent leukemia development.

**Abstract:**

For almost 30 years, the term “holobiont” has referred to an ecological unit where a host (e.g., human) and all species living in or around it are considered together. The concept highlights the complex interactions between the host and the other species, which, if disturbed may lead to disease and premature aging. Specifically, the impact of microbiome alterations on the etiology of acute lymphoblastic leukemia (ALL) in children is not fully understood, but has been the focus of much research in recent years. In ALL patients, significant reductions in microbiome diversity are already observable at disease onset. It remains unclear whether such alterations at diagnosis are etiologically linked with leukemogenesis or simply due to immunological alteration preceding ALL onset. Regardless, all chemotherapeutic treatment regimens severely affect the microbiome, accompanied by severe side effects, including mucositis, systemic inflammation, and infection. In particular, dominance of *Enterococcaceae* is predictive of infections during chemotherapy. Long-term dysbiosis, like depletion of *Faecalibacterium*, has been observed in ALL survivors. Modulation of the microbiome (e.g., by fecal microbiota transplant, probiotics, or prebiotics) is currently being researched for potential protective effects. Herein, we review the latest microbiome studies in pediatric ALL patients.

## 1. Current Status of Microbiome Analysis in Pediatric ALL

Acute lymphoblastic leukemia (ALL) is the most frequent type of pediatric cancer [1], with an incidence rate of 5.4 per 100,000 cases in patients below the age of 15 years in 2017. Incidence peaks at 2–5 years of age [1]. Three major factors increase the risk of ALL development: exposure to radiation, previous cancer treatment, and genetic disorders. A distinction is made between B- and T-cell ALL cell lineages. The most common form is B-cell lymphoblastic leukemia, which involves several genetic disorders, such as ETV6-RUNX1, BCR-ABL1, or hyperdiploidy [1]. In the last decade, microbial disbalance has been shown to influence the development of several immune diseases, including systemic lupus erythematosus, rheumatoid arthritis, and systemic sclerosis [2,3,4]. It has also been associated with oncogenesis and cancer progression in, for instance, the breast, pancreas, and white blood cells [4,5]. The influence of the microbiome on cancer development has been studied since 2012 [6], and investigations into adult ALL started two years later [7]. Although microbial disbalances have a substantial impact on several immune diseases, in cancer, it is remarkable that no relevant studies into childhood ALL were published until 2016 [8]. This might be attributed to the fact that microbiome analyses are susceptible to various problems, such as contaminations during preparation via buffers or handling, or poor processing choices due to high sequencing costs. Over the last decade, advances in technology and reductions in cost have increased microbial research interest and therefore the number of microbiome studies in leukemia [9]. Mouse experiments in the preclinical mouse model of Pax5/- and Sca1-ETV6-Runx1 mice already point to genetic predispositions altering the gut microbiome [10]. Before the onset of disease the mouse microbiome had been different between the different mouse genotypes [10]. This may further affect leukemia development and might even be used as a diagnostic tool in the future. Furthermore, based on epidemiological data, the current hypothesis of infection-triggered leukemia of the most common ALL subtypes (ETV6-RUNX1 and high-hyperdiploid chromosomes) during early childhood implies that a lack of microbial exposure in infancy can promote the emergence of this disease [11]. In 2018, stool samples of 42 pediatric patients at different timepoints of therapy were analyzed [12]. The comparison of acute myeloid leukemia (AML), ALL, and bone marrow transplant patients revealed a cancer-distinct microbiome. Specifically, a study in 2020 revealed the human microbiome as a diagnostic tool for specific cancer types [13].

Microbial composition is well adjusted to the specific conditions in different areas of the human body, depending on age, environmental lifestyle, antibiotic therapy, and diet. The symbiotic interplay between host and bacteria results in a healthy mucosa, which offers protection from overt pathogens and provides nutrients, such as short fatty acids and branched-chain amino acids, as well as the production of glycans and lactic acids (Table 1) [14,15,16,17,18,19,20]. Furthermore, the microbiome shapes and influences the immune system in a positive way by inducing innate and adaptive immune responses [4]. Studies have further revealed a profound impact on the early development of the innate immune system, referred to as trained immunity [15].

The uptake of different bacteria in early childhood, by breast feeding, for example [27], is crucial for the development of a healthy immune defense, which decreases susceptibility to various infectious pathogens and autoimmune diseases [4]. Additionally, specific commensal bacteria directly affect immune cells; for example, *Fusobacterium nucleatum* interacts with natural killer cells (NK cells) to inhibit cytotoxicity, (Table 1) [21]. Helicobacter infection leads to inflammation, T cell infiltration and apoptosis [24]. *Streptomyces carpaticus* isolated of stool samples from healthy children was incubated with cancer cells in vitro and had a killing effect on malignant cells derived from a chronic lymphocytic leukemia patient [28]. Furthermore, intra-tumor injection of *Streptomyces carpaticus* in mice also prevents cancer cells infiltration of the light tight and so far, metastasis [28].

Interestingly, patients with acute myeloid leukemia and higher baseline levels of *Porphyromonadaceae* have been shown to be protected against infections [29], and the clinical outcome of allogeneic hematopoietic stem cell transplantation can be predicted by specific enteric biomarkers [30]. In contrast, no specific oral or gut microbiota has yet been identified as playing a role in ALL. 

## 2. Microbiome in ALL

### 2.1. Microbiome at Time of Diagnosis

**Figure 1:**
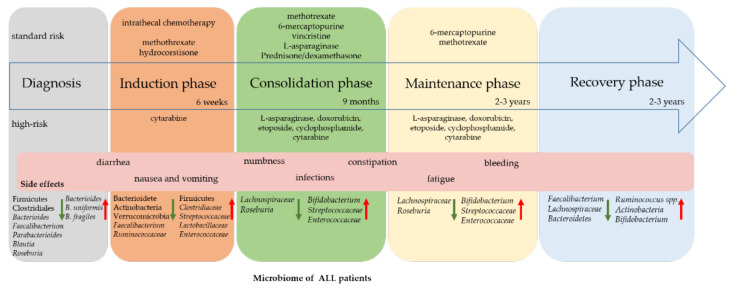
Microbial shift and side effects during therapy. During cancer therapy microbial composition shifts depending on medical treatment. Up (red arrow) and downregulated (green arrow) bacteria are listed from time-point of diagnosis, induction, consolidation, maintenance, and recovery phase. Side effects occur between all phases and cannot be assigned to a specific time. Therapy is divided into standard and high-risk with differences in therapeutical treatment.

Several studies investigated the microbiome disbalance during and after the onset of ALL (Table 2). While the symbiotic interaction between microbiome and host might become important as a future diagnostic tool, it may also lead to therapy strategies that increase medical treatment success and prevent side effects. Knowing that the microbiota composition prevents pathogen colonization, interacts with the immune system, and enhances barrier function against bacterial blood stream contamination [4], it is important to understand how an increase or decrease in bacteria affects treatment strategy (Figure 1). 

Marker genes or whole DNA content of the many bacterial, viral, fungal, and archaeal species that make up a single microbiome are nowadays routinely sequenced via short read platforms like Illumina or in growing numbers with long read platforms like Pacbio or Oxford Nanopore. Alpha diversity is a measure for the complexity of a community in a single sample and is quantified, e.g., by the sheer number of different bacterial “species”. The term species is a taxonomic rank, which remains to be assigned to the sequencing data and we should better use the terms “Operational Taxonomic Unit” (OTU), “Amplicon Sequencing Variant” (ASV) or “feature” instead of “species” in this context. Other alpha diversity metrics assess heterogeneity (Shannon index) or additionally consider evolutionary relationships between features (Faith’s Phylogenetic Diversity). To express differences between two microbial communities, various metrics of beta diversity have been developed, e.g., Jaccard, which quantifies the number of shared features between two samples, or Bray Curtis, which is bases on Jaccard but weighs features with their relative abundance in both samples. Similar to Faith’s PD, UniFrac incorporates distances. Much cheaper short read platforms limit sequencing to only parts of the full 16S rRNA marker gene. Popular choices are V1-V2, V3-4, or V6, but all come with significant biases in phylogenetic resolution, i.e., different species have the same sequence, and render meta analyses across different studies almost impossible (Table 2). 

Comparing human stool samples with different 16S regions as targets showed region-specific differences, indicating poorly classified sequences in phyla or at the genus level [43]. Keeping that in mind, microbial analysis at the time of leukemia diagnosis is rare but necessary to identify bacteria that support leukemia development early diagnosis and prophylactic strategies to improve health status during chemotherapy.

#### 2.1.1. Oral Microbiome 

The microbiome harbored in the oral cavity is the second largest and second most diverse in the body. Disbalance might result in leukemic manifestations like paleness of oral mucosa, discoloration of gums, gingival petechiae, ulcerative necrotic lesions [44], or mucosal ulcers [45]. Impairment of the mucosa as the first natural protective barrier leads to subsequent diseases. Knowing the impact of ALL on the oral cavity at diagnosis and during chemotherapy, it is astonishing that only one study has investigated the microbiota at time point of diagnosis [31].

In some cases, patients suffer from tooth and jaw pain, gingival swelling and loose teeth, all of which might be influenced by a disturbed microbiome [31]. Oral samples from 13 newly diagnosed ALL patients and 12 healthy control children were collected 2 h after breakfast [31]. Of the 12 identified phyla, Proteobacteria, Firmicutes, Fusobacteria, Actinobacteria, and Bacteroidetes were the most prominent in both patients and healthy children. Comparing ALL patients and healthy controls, Firmicutes and Fusobacteria were significantly different. ALL patients had an increased abundancy of Firmicutes, while Fusobacteria abundance was decreased. *Granulicatella* and *Veillonella*, both belonging to Firmicutes, were more abundant in ALL patients [31]. Taken together, ALL patient samples displayed a reduced microbial diversity and lower richness, indicating a disbalance that might increase infection risks.

Notably, the above study differed from others in its sample collection methods, since researchers extracted supragingival plaque from teeth instead of the cheek or tongue mucosa, so further investigation focusing on the oral microbiome is required. 

#### 2.1.2. Gut Microbiome

Additional studies have been published focusing on the gastrointestinal microbiome as a potential factor in long-term effects after therapy (Table 2). Stool samples of acute B-cell leukemia and matched healthy controls show an increased abundance of *Faecalibacterium,* Bacterioides, or Parabacterioides, but a significantly reduced alpha diversity in patient samples [8,32]. In control groups, taxa such as *Roseburia* [8,33] and Firmicutes [32] show higher abundance instead. Interestingly, diarrhea-causing Clostridiales are less abundant in ALL children, as are *Lachnospiraceae,* whose members, including *Roseburia* and *Blautia*, produce short-chain fatty acids with an anti-inflammatory benefit (Figure 1) [8,32,46]. 

During the initial manifestation of the disease, some ALL patients receive antibiotic treatment because of their increased susceptibility to infection. Administration of antibiotics temporarily perturbs microflora, sometimes even permanently. Microbiota diversity significantly decreases in ALL patients treated with antibiotics in the one-month period prior diagnosis [8,32]. Comparing the microbiome composition of ALL patients treated with short- and long-term medication prior to chemotherapy identified a further change in alpha diversity, with a decreased number of specific bacteria like Firmicutes and Bacteroidetes [8,32]. In ALL patients, representatives of Firmicutes were fewer and Bacteroidetes increased abundantly [32]. This ratio was also seen in antibiotic-treated ALL patients compared to untreated patients, which means the antibiotic treatment has less effect on Firmicutes/Bacteroidetes ratio in ALL patients [32]. Based on these results, Bai et al. considered Bacteriodales and *Enterococcaceae* to be representative of the phylum Firmicutes as a possible biomarker for ALL, but only in children without antibiotic treatment [32].

In 2020, the largest cohort to date, 70 newly diagnosed ALL patients, was recruited at time of diagnosis [33]. Ultimately, stool samples of 60 patients with ALL and 23 healthy children were analyzed, after excluding those treated with antibiotic or probiotic supplements. While there was no difference in the alpha diversity, this study also verified increased Bacterioides species in the beta diversity. Specifically, *Bacterioides uniformis* and *Bacteroides fragiles* were significantly increased in pre-chemotherapy ALL patient samples [41]. Variations in the alpha and beta diversity may be caused by analyzing different regions of the 16S rRNA. In the first studies, V1-V3 regions were amplified, while Bai et al. went further into the V3-V4 hypervariable regions, V1-V9, and latter V4 region (Table 2) [8,31,32,33]. 

### 2.2. Changes during Therapy

**Figure 2:**
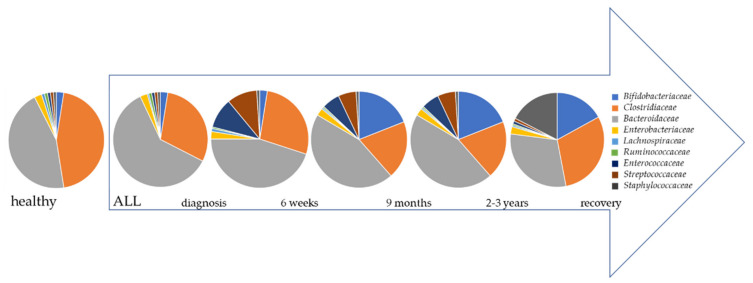
Microbial composition shifts during therapy phases. At timepoint of diagnosis composition already differs from ALL patients compared to healthy children. *Clostridiaceae* and *Bacteroidaceae* are predominant species in healthy children [31,48]. At time of diagnosis *Bacteroidaceae* abundance prevails in ALL patients while *Clostridiaceae* and *Lachnospiraceae* decrease [31]. During the first 6 weeks of therapy namely induction phase, *Ruminococcacea* decrease, unlike *Streptococcaceae* and *Enterococcaceae*, whose abundances increase [35,36,38]. While bacterial load decreases in general within consolidation (9 months) and maintenance phase (2–3 years) the abundance of *Lachnospiraceae* and *Clostridiceae* decreases drastically, whereas spectrum of *Bifidobacteriaceae, Streptococcacea,* and *Enterococcaceae* emerged [35]. At the end of therapy microbial composition significantly differs from healthy children. ALL children still show an increase of *Bifidobacteriaceae* and *Staphylococcaceae*, and a decrease of *Clostridiaceae* and *Bacteroidaceae* [41]. This figure is a schematic representation and displays trends, not exact numbers.

Early symptoms of childhood ALL, such as anemia-derived fatigue, fever, infection, and even easy bleeding, are often a result of the reduced number of blood cells. Therapy starts directly after diagnosis, with chemotherapy given in 3 phases over 2–3 years (Figure 1). Chemotherapy leads to a shift in bacterial composition and a decrease of the white blood cell counts; together this often leads to infection induced by bacteria. In a study with 409 newly diagnosed ALL patients, 1313 microbiologically derived infections were documented during therapy [47], and it was hypothesized that intestinal microbial changes caused infectious side effects. Although the damage done to the microbiome by chemotherapy has long been known, systematic investigation of altered microbial composition in ALL patients only started in 2016. Stool samples were collected from 28 newly diagnosed ALL patients at different points of treatment and from 23 controls [8]. Comparing the microbiome before and after treatment, the diversity of composition was significantly reduced, with a specific reduction of *Lachnospiraceae* and *Roseburia* in patient samples. A larger study from Hakim et al. investigating the different time points analyzed 199 ALL patient stool samples at three different points during chemotherapy [35]. Interestingly, comparing the different time points with each other, no significant difference was found in the mean diversity, and furthermore it “recovered” to baseline, but the microbial diversity significantly decreased after chemotherapy, with a different bacterial composition (Figure 2). 

During therapy, *Bacterioidetes*, *Faecalibacterium*, *Ruminococcaceae*, Actinobacteria, and Verrucomicrobia significantly decreased, while other taxa, *Clostridiaceae*, *Streptococcaceae*, *Lactobacillaceae*, *Enterococcaceae,* and Firmicutes, increased [35]. Two more studies have underlined the reduced microbial diversity related to chemotherapy treatment, analyzing in each case 32 and 51 newly diagnosed ALL patients with 13 different time points within a year and 5 time points within a month, respectively [36,38]. 

It seems that beta diversity of the family *Ruminococcaceae* decreased [35], but specific species (e.g., *Ruminococcus gnavus* and *Ruminococcus torques)* tended to increase as a result of chemotherapy and were elevated after a year [36]. However, not only was the beta diversity diminished, but the alpha diversity in patient samples was also reduced within one month after chemotherapy, and then increased on day 29 [38]. 

Five years later, based on their initial study, Rajagopala et al. focused on prophylactic antibiotic treatments during therapy to prevent side effects [36]. Likewise, De Pietri et al. directly pointed out the interplay between bacterial species and specific proteins of systemic inflammation and enterocyte loss [38]. 

The first examination of temporal changes in the gut microbiome was conducted via a longitudinal observation study, with samples collected from seven ALL patients before, during and after chemotherapy and from controls [41]. Antibiotic treatment before chemotherapy made no difference that might explain the observed large inter-individual variability in ALL patients compared to healthy children. Nevertheless, Bacteroidetes was significantly enriched before chemotherapy and although its abundance decreased after therapy, it was still higher than in control samples [41]. Abundance of Firmicutes and Actinobacteria, on the other hand, increased following chemotherapy to a level similar to that of healthy controls. Five genera stood out with a lower abundance after therapy: *Bacteroides* and *Prevotella*, belonging to the phylum Bacteroidetes; *Fusobacterium*; and *Atopobium* and *Corynebacterium* from Fusobacteria and Actinobacteria, respectively. Interestingly, only *Bifidobacterium* (Actinobacteria) was significantly higher in the post-chemotherapy samples. This commensal occurs immediately following birth [49] and was described to be able to use human milk oligosaccharides, as well as other carbon sources [50]. Furthermore, it has a protective role in preventing intestinal inflammation in infants since it prevents an increase of Proteobacteria which are associated with dysbiosis and negative health outcomes [50].

### 2.3. Complications, Infections

Antibiotic treatment and chemotherapy have detrimental effects on microbial composition. Febrile neutropenia, diarrheal, and therefore systemic inflammation and enterocyte loss often occur during therapy [35,38]. For instance, pathogenic bacterial overgrowth negatively affects the mucosa and thus the associated immune system [51,52]. Bloodstream infections occur due to an ineffective natural barrier against the invading microorganisms [52], indicating that a healthy microbiome not only provides nutrition and a natural barrier, but it also influences the outcome of medical treatment. 

Firmicutes and Bacteroidetes are the prominent phyla in the gut microbiome. The ratio between Firmicutes (decrease) and Bacteroidetes (increase) has been introduced as an indicator of a dysregulated microbiome [32]. The Firmicutes and Bacteroidetes change in ALL patients is independent of antibiotic treatment, but it leads to a dysregulation in healthy controls [32]. For example, *Megamonas*, a species which belongs to the phylum Firmicutes and is correlated with inflammation, is more abundant in antibiotic-treated ALL patients [32,53]. 

During chemotherapy, patients suffer from bloodstream infection, diarrheal illness, or febrile neutropenia and can also develop oral diseases. In a cohort of 39 ALL-diagnosed and 39 healthy children, only ALL patients developed oral mucositis and candidiasis (15.4% and 2.6%, respectively) [40]. *Lactobacillales* belonging to the phylum Firmicutes were more abundant in ALL patients. The increase in *Lactobacillus* strains [40] is associated with reduced wound-healing capacity [54]. Dental cavities and gingivitis also occurred more frequently in ALL patients (69.4% vs. 38.5%) [40].

In a cohort of 199 treated children diagnosed with ALL, 13% suffered a bloodstream infection, 61% a febrile neutropenia and 37% a diarrheal illness [35]. *Enterococcus*, *Staphylococcus* and *Enterobacter* were found in the bloodstream, corresponding to an increase in *Enterococcaceae* or *Streptococcaceae* during therapy [35]. The increase in *Streptococcaceae* was also associated with diarrheal illness and *Enterococcaceae* with developing febrile neutropenia. Investigation in a rat model showed that diarrhea was accompanied by a decreased presence of commensal protective anaerobic species like *Ruminococci* and oxygen-tolerant *Streptococci* [55]. This trend is also seen in ALL patients, but the impact of single therapeutics on the microbiome is less investigated in humans. One such compound is methotrexate, a chemotherapeutic drug commonly used in the treatment of ALL (Figure 2) [56]. Its influences on the microbiome have been investigated in mouse and rat models, as well as in ALL-diagnosed children [34,55,57]. After high-dose methotrexate therapy in ALL patients, *Bifidobacteria*, *Lactobacillus,* and *Escherischia coli* (*E. coli)* abundancy decreased [34]. *E. coli* protects mice from intestinal inflammation caused by *Salmonella typhimurium* infection [25]. Furthermore, it was described to prevent intestinal colonization by *Pseudomonas aeruginosa* [52]. *Lactobacillus* was described to reduce inflammation by regulation of regulatory T cells, which secrete the anti-inflammatory cytokine IL-10 [58]. Surface-associated molecules from *Bifidobacteria* act by activating pattern recognition receptors (PRRs) on immune cells to elicit an immunomodulatory response [59]. In a mouse model, mucosal damage was accompanied by inflammatory cell infiltration, as well as gradually reduced diversity [57]. Comparable with human studies, Bacteroidetes abundancy was reduced while, contrary to ALL patients during therapy, * Lachnospiraceae* was increased [57]. During the consolidation phase, patients are commonly treated with corticosteroids (e.g., prednisone or dexamethasone) in addition to methotrexate (Figure 1), which are known to affect the microbiome directly. For instance, dexamethasone application leads to an increase in *Bifidobacterium* and *Lactobacilli*, and a decrease in *Mucispirillum*, while treatment with prednisone has an increasing effect on the abundance of *Streptococci* and *Bifidobacteria* [60]. An increase in *Faecalibacterium* occurs with enhanced occludin and E-cadherin expression [60]. Intestinal microbiota also interfere with treatment by degrading corticosteroids into inactive compounds. *Clostridium scindens* has been shown to metabolize prednisone in a mouse model [61], but this effect has not yet been investigated in ALL patients. The cytostatic drug 6-mercaptopurine is metabolized to 6-thioguanine, which leads to cell cycle arrest and apoptosis [62]. Interestingly, it has been shown that *E. coli* also metabolizes 6-mercaptopurine to active thioguanine nucleotides without host enzymatic reaction [60]. *Bacteroides fragilis* and *Enterococcus faecalis* provide enzymes that are necessary for conversion into an active compound, such as hypoxanthine-guanine phosphoribosyl transferase (HGPRT) or guanosine monophosphate synthetase (GMPS) [60,63], indicating the importance of microbial composition. Additional studies are available for high-risk therapeutics like doxorubicin, etoposide, and cyclophosphamide. In a mouse model, administration of doxorubicin increased epithelial barrier permeability, which led to bacterial translocation and, consequently, mucosal damage [64]. In the colon, *Raoultella planticola*, a member of the *Enterobacteriaceae* family, inactivates doxorubicin via an anaerobic-dependent deglycosylation pathway [65]. Cyclophosphamide treatment leads to increased intestinal permeability and, as a consequence, to bacterial translocation [66]. Additionally, the predominance of potentially pathogenic species belonging to *Enterobacteriaceae*, *Enterococcaceae*, and *Pseudomonaceae* can occur [67]. Viaud et al. demonstrated in a mouse model that Gram-positive members of Firmicutes like *Lactobacillus johnsonii*, *Lactobacillus murinus*, or *Enterococcus hirae*, were detected in the mesenteric lymph nodes and spleen, which led to a stimulation of TH17 and memory TH1 immune responses, thus possibly enhancing anticancer immune responses [68]. 

Furthermore, antibiotic-treated ALL patients who suffered an infection within the first 6 months of therapy had a lower phylogenetic diversity in 9 out of 16 cases [39]. Although the infection rate was higher in females, no difference was found between the sexes via Faith‘s phylogenetic diversity. Comparing microbial composition between ALL patients with and without infection, *Faecalibacterium prausnitzii* was more abundant in non-infected patients and completely absent in patients with infection and antibiotic treatment [39]. This commensal produces butyrate and reduces proinflammation by blocking the inflammatory cytokine Interleukin-8 [23]. Additionally, *Lachnospiraceae* and *Roseburia*, species also known to produce butyrate [69], are reduced in ALL patients [8], which is suggested to increase the risk of mucositis chemotherapy dependently. On the other hand, the abundance of the non-fermenting bacterium *Brevudnimonas diminuta,* which can be resistant to many different antibiotics, is increased in ALL patients with infectious events [39,70]. In order to investigate factors that cause intestinal mucositis, which can be detected via plasma citrulline levels, stool samples from 51 children were analyzed at different chemotherapy time points [38]. In children with AML, mucositis could be diagnosed using inflammation and cell loss, by measuring Interleukin-8 and plasma citrulline, respectively [71]. Plasma citrulline is produced by enterocytes [38]. Lower levels of citrulline correspond to cell loss and thus to tissue damage and inflammation [72]. Mucositis was also introduced as marker for an increased risk of bacteremia in ALL [72]. Furthermore, plasma C-reactive protein level acts as an indicator for inflammation, and its direct increase at sites of infection was also correlated with the increase in specific bacteria at different therapy time points [38]. Alpha diversity was decreased until day +22, and was associated with a low plasma citrulline and a high CRP level during mucositis episodes [38]. Interestingly, high citrulline and low levels of CRP were linked to an increased abundance of *Lachnospiraceae* bacteria, which are involved in the biosynthesis of acetate and butyrate. On the other hand, high CRP levels and low citrulline concentrations were associated with an increased abundance of *Enterococcus* and maximum mucositis severity and inflammation [38]. Low microbial diversity that decreases within the first month is linked to an increase in enterocyte loss and systemic inflammation.

### 2.4. Reconstitution after Therapy

Despite the restoration of normal health after the completion of chemotherapy, several studies have found changes in the microbial gut profile of post-treatment ALL patients to be ongoing [41]. At least 3 months after treatment, the complete bacterial composition of ALL patients remained clearly distinct from healthy controls (Figure 2) [37]. 

Although no significant differences in alpha diversity could be detected, differences in composition and abundance of some bacteria were still identified [37]. Thomas et al. were able to detect differences between the gut microbiome of ALL survivors (at least 1 year after treatment completion) and that of healthy siblings [42]. Even though no statistically significant differences in alpha or beta diversity could be detected, depletion of members of the *Lachnospiraceae* and *Ruminococcaceae* families, including *Faecalibacterium*, was remarkable [42]. One further study showed that a year after the start of chemotherapy treatment, the gut microbiota had stabilized, but species richness never fully recovered [36]. In particular, abundances of, e.g., *Ruminococcus gnavus* and *Ruminococcus torques* were still increased one year after chemotherapy [36]. This suggests that the composition of the gut microbiota is modulated, with some species being substantially altered.

Long-term adult survivors of pediatric ALL who completed therapy at least 5 years before the study by Chua et al. showed reduced microbial diversity compared to healthy controls, with a notable enrichment of *Actinobacteria* and depletion of *Faecalibacterium* [41]. Furthermore, in these individuals, increased T-cell activation and chronic inflammation was observed, suggesting a correlation between dysregulated microbial taxa and immune dysregulation [41]. The increased infection risk in survivors [73], a high prevalence of chronic health conditions [74,75] and an elevated risk of mortality and morbidity have been investigated [76]. Thus, microbial dysregulation due to the influences of chemotherapy and antibiotics during ALL treatment may have long-term effects on the development of other diseases, such as obesity or diabetes, in adult survivors of pediatric ALL [41,42]. 

## 3. Outlook: Modulation of the Microbiome

Due to the described microbiome changes in ALL patients at diagnosis, over the course of chemotherapy and persisting even after several years, modulation of the microbiome is the subject of current research related to its potential protective effects against leukemia development, and its effects on the course of treatment and patient outcome. 

Possible strategies to prevent side effects, such as fecal microbiota transplantation (FMT), administration of probiotics or prebiotics, are also under investigation. 

FMT is the transfer of fecal substance from a donor to a recipient with the intent to change the recipient’s gut microbiota and thus confer a health benefit [77,78]. In recent studies, FMT has been used to treat *Clostridium difficile* (*C. difficile*) infection, with success rates of up to 85% [79,80]. However, for example in inflammatory bowel diseases FMT did not seem to be as effective as in the treatment of *C. difficile* infection [77]. A possible explanation could be that the pathophyisiology of these diseases is multifactorial [77]. Clinical trials for the treatment of inflammatory bowel diseases in pediatric patients are ongoing [81]. However, no trials focused on hematologic malignancies in children are currently registered. Thus far, there is only data for mouse experiments and case reports for adult patients [78,82,83]. In mice, FMT was able to restore the gut microbiome after disruption via antibiotic and chemotherapy administration [83]. In adult patients with blood disorders who harbored antibiotic-resistant bacteria, FMT was performed to eradicate those bacteria, with success rates of 75% for complete decolonization and 80% for partial decolonization [82]. However, with this procedure a theoretical risk for infections exists since the exact microbial composition of the transplant is not always known [77]. Furthermore, the effects of this procedure in immunocompromised patients need to be further investigated. Standardization of donor screening and selection, as well as sample preparation, route of administration and dosing schedules is needed to be able to systemically examine the effects and risks of this treatment [77,78,80]. Due to a lack of data for pediatric ALL patients, optimal approaches for immunosuppressed patients still need to be determined, in order to minimize the risk of infections and procedure-related complications [78]. In particular, the crucial organisms have to be identified and the optimal donors have to be recruited and screened for potential pathogenic microbes in order to guarantee the highest possible level of safety, since the composition of the donor’s fecal substance will affect the outcome of the procedure.

Another possibility for modulation of the microbiome is the use of probiotics. Probiotics are defined as “isolated viable organisms administered to confer a health benefit on the host” [78], which can be taken up as part of the diet (e.g., yoghurt, kefir) or in capsules as medication [84,85]. For pediatric patients, only a few studies have been published to date investigating the effect of probiotics. In two studies by Reyna-Figueroa et al., postchemotherapy complications like gastro-intestinal side effects were diminished in children with ALL through the use of probiotics, namely *Lactobacillus rhamnosus GG* [86,87]. However, complications due to infection caused by probiotics have been reported in adult patients [88,89], due to the presence of live organisms that represent a risk for immunocompromised patients. Thus, the use of probiotics must be applied cautiously, with further studies warranted. Precision medicine may be needed, with characterization of the patients’ gut microbiota, the underlying disease and side effects, in order to guarantee the most effective probiotic treatment.

While FMT and probiotics use live organisms to modulate the microbiome, prebiotics are “substrate[s] that [are] selectively utilized by host microorganisms conferring a health benefit” [90], such as starches or the galacto-oligosaccharides contained in milk [78]. These substances are fermented by microorganisms to produce short-chain fatty acids (SCFAs) like butyrate, which influence immune cell signaling and chemotherapy toxicity and efficacy, among other factors [16,78]. SCFAs have been shown to modulate regulatory T-cell responses in the intestine, resulting in the suppression of pro-inflammatory immune cells [17,22,78,91,92]. Thus, supplementing ALL patients with prebiotics may result in a favorable outcome and diminish side effects during therapy. However, further studies are needed to better understand the interactions between diet, expansion of microorganisms and clinical outcomes [78]. 

## 4. Conclusions

This review gives an overview of microbiome changes in patients with childhood ALL at the time of diagnosis and during treatment, with possible implications for complications, and persisting differences after the completion of therapy. At the onset of disease, reduced diversity in ALL patients is already observed in the oral and gut microbiomes. Further reduction of diversity occurs during treatment, due to the administration of chemotherapeutics and antibiotics. A disturbed microbiome also has implications for side effects during treatment, with dominance of *Enteroccocaceae* being predictive of infections. Changes to the microbiome, including depletion of *Faecalibacterium*, can be detected up to several years after completion of treatment, with possible implications for long-term health. However, even with all those studies it remains unclear whether the observed alterations are a causal link for ALL development or due to immunological alterations preceding the emergence of ALL. Monitoring of large pediatric cohorts could be useful to provide direct evidence if the genotype already determines microbial composition, even without the onset of disease.

Taking all of this together, there is a clear need for precise characterization and modulation of patients’ microbiomes at each time point during therapy, in order to better understand the microbial influence on leukemogenesis, minimize side effects and improve treatment efficacy.

## Figures and Tables

**Table 1:** Bacterial influence on nutrition and immunity.

Phylum	Genera	Species	Nutrient	Immunity	Refs.
Fusobacteria		*F. nucleatum*		NK	[21]
Firmicutes	Clostridium	Cluster XIVa/IV	butyrate	Treg	[22]
	Eubacterium		lactic acid		[19]
	Faecalibacterium	*F. prausnitzii*	butyrate	TH17/Treg	[23]
	Roseburia		SCFAs	Treg	[17]
	Ruminococcus		butyrate	Treg	[22]
	Enterococcus		lactic acid		[18]
Bacterioidetes	Bacteroides		glycans		[20]
	Prevotella		carbohydrates		[16]
Actinobacteria	Bifidobacterium		SCFAs		[16]
Proteobacteria	Helicobacter			TH1	[24]
	Escherischia			protective against *Salmonella typhimurium* and *Pseudomonas aeruginosa*	[25,26]

**Table 2:** Microbiome studies in pediatric ALL patients.

Time Point	Sample Type	Patients	Controls	Time Points	Seq. Region	Refs.
At time of diagnosis	Oral	13	13	1	V1-V3	[31]
	Stool	23+5	23	4	V1-V3	[8]
	Fecal	30	33	1	V3-V4	[32]
	Stool	58	23	1	V1-V9	[33]
During therapy	Stool	36	36	patients = 3; controls = 1	N/A	[34]
	Stool	199	0	4	V1-V3	[35]
	Stool	32	25	13	V4	[36]
	Anal swabs	7	7	3	V4	[37]
	Stool	51	19	patients = 5; controls = 2	V3-V4	[38]
Complications during therapy	Stool	42 (15 ALL)	0	1	V3-V4	[12]
	Stool	199	0	4	V1-V3	[35]
	Stool	16	0	2	V4-V5	[39]
	Stool	51	19	patients = 5; controls = 2	V3-V4	[38]
	Oral	39	39	1	V1-V3	[40]
After therapy	Anal swabs	73	61	1	V4	[41]
	Stool	32	25	13	V4	[36]
	Stool	38	16	1	V4	[42]
	Anal swabs	7	7	3	V4	[37]

## Data Availability

Data sharing not applicable. No new data were created or analyzed in this study. Data sharing is not applicable to this article.
